# Treatment with Insulin Analog X10 and IGF-1 Increases Growth of Colon Cancer Allografts

**DOI:** 10.1371/journal.pone.0079710

**Published:** 2013-11-18

**Authors:** Henning Hvid, Marie-José Blouin, Elena Birman, Jesper Damgaard, Fritz Poulsen, Johannes Josef Fels, Christian Fledelius, Bo Falck Hansen, Michael Pollak

**Affiliations:** 1 Diabetes Research Unit, Novo Nordisk A/S, Maaloev, Denmark; 2 Lady Davis Institute for Medical Research, Jewish General Hospital, Montreal, Quebec, Canada; Virginia Tech, United States of America

## Abstract

Obesity and type 2 diabetes are associated with an increased risk for development of certain forms of cancer, including colon cancer. The publication of highly controversial epidemiological studies in 2009 raised the possibility that use of the insulin analog glargine increases this risk further. However, it is not clear how mitogenic effects of insulin and insulin analogs measured *in vitro* correlate with tumor growth-promoting effects *in vivo*. The aim of this study was to examine possible growth-promoting effects of native human insulin, insulin X10 and IGF-1, which are considered positive controls *in vitro*, in a short-term animal model of an obesity- and diabetes-relevant cancer. We characterized insulin and IGF-1 receptor expression and the response to treatment with insulin, X10 and IGF-1 in the murine colon cancer cell line (MC38 cells) *in vitro* and *in vivo*. Furthermore, we examined pharmacokinetics and pharmacodynamics and monitored growth of MC38 cell allografts in mice with diet-induced obesity treated with human insulin, X10 and IGF-1. Treatment with X10 and IGF-1 significantly increased growth of MC38 cell allografts in mice with diet-induced obesity and we can therefore conclude that supra-pharmacological doses of the insulin analog X10, which is super-mitogenic *in vitro* and increased the incidence of mammary tumors in female rats in a 12-month toxicity study, also increase growth of tumor allografts in a short-term animal model.

## Introduction

Obesity and type 2 diabetes are associated with an increased risk for certain forms of cancer, such as breast, pancreatic and colon cancer [1]–[5]. Highly controversial epidemiological studies suggested that therapeutic use of the insulin analog insulin glargine was associated with an increased risk for development of cancer [6], [7], but the ORIGIN trial recently provided strong evidence that this is not the case [8]. However, the epidemiological studies published in 2009 and the following discussions highlighted the importance of the pre-clinical safety assessment of insulin analogs. Furthermore, the reassuring results concerning glargine do not diminish the prior evidence for an association between type 2 diabetes and increased risk or worse prognosis of certain cancers, including colon cancer, and the mechanisms behind this association remains an important topic.

In this context, the insulin analog X10 is an interesting ligand. X10 was developed as a fast-acting insulin analog by substitution of histidine at position B10 with aspartic acid [9]. This single amino acid substitution increased the binding affinity of X10 to the IGF-1 receptor (IGF-1R) and insulin receptor (IR) 3- to 5-fold and 2-fold, respectively [10], [11]. Furthermore, X10 has decreased off-rate from the IR compared to native human insulin (HI) which results in sustained signalling from the IR [12]. Recently, it was shown that X10 results in proportionally stronger activation of phosphorylation sites in the juxta-membrane and kinase domains of the IR than the C-terminal domain [13]. These receptor binding and –activation characteristics gives X10 a 3–15 fold higher mitogenic potential than HI *in vitro* [14] and in a 12-month chronic toxicity studies supra-pharmacological doses of X10 increased the incidence of spontaneous mammary tumors in female Sprague Dawley rats [15]. Further development of X10 for clinical use was therefore discontinued, but the mechanisms behind the increased tumor incidence have never been fully clarified (see [16] for a detailed review).

Previous animal studies using genetic or diet-induced models of obesity or diabetes have correlated hyperinsulinemia with increased formation of chemically induced preneoplastic lesions in colon [17], [18], growth of chemically induced colon tumors [19], [20] as well as growth of murine cancer cell allografts [21]–[25]. In rat models with chemical induction of cancer, treatment with insulin enhanced growth of azyoxymethane-induced colon tumors [26] and 7,12-dimethylbenz(a)anthracene-induced mammary tumors [27]. It has also been shown that constant infusion of insulin to rats for 12 h increased proliferation of colon epithelial cells [28], and supra-pharmacological doses of HI or glargine for 18 weeks increased proliferation of colon epithelial cells and formation of preneoplastic lesions, but did not result in tumor formation [29]. In safety studies, which traditionally are performed as carcinogenicity studies or chronic toxicity studies of 6–24 months duration in mice or rats [30], no increased tumor incidence was observed in Sprague Dawley rats and NMRI-mice after treatment with relatively low doses of glargine and HI for up to 24 months [31]. However, as mentioned above, treatment with high doses of X10 for 12 months increased the incidence of mammary tumors in the mammary tumor-prone female Sprague Dawley rats [32]. While the recommendations for safety studies of insulin and insulin analogs are based on well-validated scientific practice, originally developed for studies of mutagens, the existing data suggests that a tumor growth-promoting effect of insulin and insulin analogs is a more relevant concern, than concern for increased tumor initiation, via an increased mutation rate caused by an increased proliferation, as also suggested previously [33]. The cost-effectiveness of screening programs for different forms of cancer emphasizes that it is not uncommon for adults, including diabetics, to have undetected premalignant early cancers [34], [35]. It is therefore relevant to explore how treatment with insulin and insulin analogs influence the behaviour of existing cancers, and to do this in animal models of diabetes or obesity combined with insulin resistance, since these factors are known risk factors for cancer development.

The aim of the study was to examine the possible tumor growth-promoting effect of treatment with HI, X10 and IGF-1 in a murine colon cancer allografts model (MC38 cells) established in mice with diet-induced obesity (DIO) and insulin resistance. We therefore characterized the MC38 cell line used for the allograft studies extensively and performed a series of animal experiments to obtain a reliable estimate of the possible tumor growth-promoting effect of HI, X10 and IGF-1 *in vivo*.

## Materials and Methods

### Animal Experiments

To examine the effect of treatment with HI, X10 and IGF-1 on growth of MC38 tumor allografts we performed five identical animal experiments. We focused on different endpoints in these animal experiments and monitored tumor growth in all experiments, see Table 1. The animal experiments were performed as described recently [36]. In brief, male C57BL/6 mice were purchased from Jackson Laboratories at age of 18 weeks where the mice had been maintained on a high-fat diet (rodent diet with 60 kcal% fat, D12492, Research Diets, Inc., New Brunswick, NJ, USA) since age of 6 weeks. For characterisation of the metabolic phenotype in DIO-mice (see Table 2), metabolic parameters were also measured in age-matched lean mice fed a control diet, (Rodent diet with 10 kcal% fat, D12450B, Research Diets) included in three of the experiments. Animal care and treatments were conducted in accordance with established guidelines and protocols approved by the Lady Davis Institute (protocol # 5951) and McGill University’s Animal Ethics Committee. Mice were housed one mouse per cage with ad libitum access to tap water and high- or low fat diet, respectively, throughout the studies. The temperature in the animal rooms was maintained at 20–25°C with a light/dark cycle of 14/10 hours. Mice were acclimatized for 7–10 days before experimental procedures were initiated. At experimental day 0, 2.0×10^5^ (experiment A) or 5.0×10^5^ (all other experiments) MC38 cells, suspended in 100 µl phosphate-buffered saline (PBS), were injected subcutaneously (sc) in the right flank of the mice. Subsequently, each mouse was injected sc twice daily with either vehicle (aqueos solution containing 7 mM phosphate, 150 mM glycerol, 22 mM NaCl and 30 mM phenol, pH 7.4), recombinant human insulin 600 nmol/kg, insulin analog X10 (insulin analog B10Asp) (Novo Nordisk A/S, Copenhagen, Denmark) 600 nmol/kg or recombinant human IGF-1 (Increlex, IPSEN Pharma GmbH, Ettlingen, Germany) 600 nmol/kg. The size of the tumor allografts was measured three times per week and the volume calculated using the following formula: length × width^2^ × 0.52. Based on the tumor volume data for each mouse, we calculated the area under the tumor growth curves (tumor growth AUC). At termination of the experiments, mice were anesthetized with isoflurane. Blood was collected by cardiac puncture and immediately after euthanasia by cervical dislocation, samples of the liver, colon, gastrocnemius muscle and the MC38 cell tumor allografts were dissected out and snap-frozen in liquid nitrogen for later preparation of tissue lysate.

**Table 1:** Overview and aims of the animal experiments.

Experiment	Aim(s)
A	Monitor tumor growth
B	Monitor tumor growth
C	Monitor tumor growth
	Examine signalling in tumor allograft
	Examine PK and PD
D	Monitor tumor growth
	Examine PK and PD
E	Monitor tumor growth
	Examine PK and PD

**Table 2:** Mean values ± SEM of selected metabolic parameters in DIO-mice and lean, age-matched controls.

	DIO-mice	Lean mice
Body weight (g)	42.0±0.4 (n = 102)***	30.1±0.3 (n = 73)
Insulin levels (ng/ml)	6.91±0.35 (n = 79)***	1.59±0.10 (n = 52)
HbA1C (%)	5.3±0.08 (n = 18)***	4.9±0.04 (n = 6)
Liver triglyceride content (µg/mg tissue)	47.8±6.3 (n = 12)**	13.5±1.7 (n = 12)
AUC glucose (mmol/L × 120 min)†	2851.6±184.4 (n = 9)*	1985.1±76.5 (n = 9)
Insulin sensitivity index††	0.42±0.05 (n = 6)*	1.62±0.29 (n = 6)

† Area under blood glucose curves during a glucose tolerance test was calculated as described previously [52].

†† Whole-body insulin sensitivity indices during a glucose tolerance test was calculated as described in a previous study [43].

* , ** and *** indicate *P*<0.05, <0.001 and <0.0001, respectively, when the difference between DIO-mice and lean mice was analyzed (student t-test).

### Blood Glucose and HbA1C

To monitor the pharmacodynamic effect of treatment with HI, X10 and IGF-1, blood glucose was measured before treatment and 15 min, 1 h, 2.5 h and 6 h after treatment. Blood was collected by puncture of the saphenous vein and blood glucose measured using a OneTouch Ultra Glucometer (LifeScan, Inc., Milpitas, CA, USA).

Levels of glycosylated hemoglobin (HbA1C) in the blood of DIO- and lean control mice were measured using the Tina-quant Hemoglobin A1c Gen.3 analysis kit and a Cobas 6000 instrument (Roche Diagnostics GmbH, Mannheim, Germany), according to manufactureŕs instructions.

### Collection of Plasma Samples and Measurements of C-peptide, Human Insulin, Insulin X10 and Human IGF-1

Before experiments were initiated, the systemic levels of mouse insulin were measured using a Rat/Mouse Insulin ELISA kit (Millipore Corp., Bilerica, MA, USA), according to manufacturer’s instructions. Plasma samples collected at termination of experiments were assayed for mouse C-peptide, human insulin, insulin analog X10 or human IGF-1. Mouse C-peptide was assayed with Rat/Mouse C-peptide 2 ELISA kit (Millipore), according to manufacturer’s instructions. Plasma concentrations of human IGF-1 were measured with the IDS-iSYS IGF-1 ELISA kit (IDS Immunodiagnostics, Fountain Hills, AZ, USA), according to manufacturer’s instructions. Plasma samples were analysed for native human insulin using a Luminescence Oxygen Channelling Immuno-assay (LOCI-assay), as described previously [37]. Insulin X10 was measured in mouse plasma using a wash-LOCI assay, as also described recently [38]. See Materials S1 for detailed information regarding these assays.

### Cell Culture

MC38 cells (mouse cell line described by Corbett et al. (1975) [39] and generously provided by Dr. Pnina Brodt, McGill University, Montreal, QC) and MCF-7 cells (ATCC, Manassas, VA, USA), were cultivated in DMEM medium containing 4.5 g/l glucose (Wisent Inc., Montreal, QC, Canada) supplemented with 10% (v/v) fetal bovine serum (FBS, Invitrogen) and 20 µg/ml gentamicin (Sandoz Canada, Boucherville, QC, Canada) (i.e., growth medium). Cells used in animal experiments were trypsinized, washed once in PBS and resuspended in PBS (4°C) at a concentration of 5.0 × 10^6^ cells/ml and kept on ice until sc injection in the mice. For signalling experiments MC38 cells were cultivated in growth medium for two days until 80–90% confluent, cell cultures were then rinsed once in PBS (room temperature), and starvation medium (similar to growth medium, except it contained only 0.25% (v/v) FBS and no phenol red) was added for 3 h. After starvation cells were treated with native human insulin, X10 or IGF-1 at final concentrations of 1 or 10 nM in starvation medium. Exactly 30 min after treatment, cells were rinsed once in PBS (4°C) and lysed by addition of lysis buffer, as described above. Each signalling experiment comprised two replicate samples per treatment and three independent experiments were performed. MC38 cells used for characterization of IR and IGF-1R expression were cultured, harvested and lysed as described for the signalling experiments above, except they were not starved prior to lysis.

### Cell Proliferation

Effect of test compounds on proliferation was assessed with 3-(4,5-dimethylthiazol-2-yl)-2,5-diphenyl tetrazolium bromide (MTT) assays, essentially as described previously [40]. In brief, MC38 cells were plated in 96-well plates (5,000 cells per well) in growth medium. After incubation for 1 day, cells were rinsed in PBS (room temperature), starved for 3 h and then treated with HI, X10 or IGF-1 in concentrations ranging from 0.001 to 1000 nM. Data for relative cell numbers from the three repeated experiments, each with four replicate samples per condition, were then used to fit dose-response curves using GraphPad Prism version 6.0 (GraphPad Software Inc., La Jolla, CA, USA). Cell proliferation experiments were done with MCF-7 cells as described for MC38 cells, except that 20,000 cells were plated per well. See Materials S1 and Figure S1 for detailed supplementary information regarding cell proliferation experiments.

### Preparation of Tissue and Cell Lysates and Western Blotting

Lysis of frozen tissue samples and cell cultures in Petri dishes, centrifugation of lysates, assay of protein concentration, mixing of cell or tissue lysates with 2X SDS loading buffer, denaturation, SDS-PAGE on pre-cast 4–15% gradient gels (BioRad) and transfer to 0.45 µm nitrocellulose membranes were performed as described previously [36] Prior to blotting with primary antibodies the nitrocellulose membranes were blocked by incubation in Tris-buffered saline with 0.05% (v/v) Tween (TBS-T) with 5% (w/v) bovine serum albumin (BSA) (when blotting with phosphorylation-specific primary antibodies) or TBS-T with 5% (w/v) skim milk (all other primary antibodies) for 1 h at room temperature. Primary antibodies were diluted in TBS-T with 5% (w/v) BSA and incubation was performed overnight at 4°C. The following rabbit antibodies, all from Cell Signalling Technology Inc., Boston, MA, USA, were used: anti-phospho-p70S6K (Thr389, cat. no. 9205), anti-P-S6 (Ser235/236, cat. no. 2211), anti-phospho-Akt (Ser473, cat. no. 9271), anti-Akt (cat. no. 9272), anti-phospho-MAPK (Thr202/Tyr204, Thr185/Tyr187, cat. no. 4370), anti-P-IRS-1(Ser302, cat. no. 2384), anti-IRβ (cat. no. 3025), anti-IGF-1Rβ (cat. no. 3018), anti-beta-actin (cat. no. 4967). Incubation with secondary antibody (horse radish peroxidase-conjugated goat anti-rabbit IgG (Santa-Cruz Biotechnology, Santa Cruz, CA, USA, cat. no 5401) and visualization of protein bands was done as described previously [36]. The intensity of protein bands was quantified using the software ImagePro (BioRad). In each cell signalling experiment, band intensities were normalized to the mean of the vehicle-treated samples. IR and IGF-1R bands in the liver, muscle colon and MC38 cell allografts were normalized to mean band intensities in liver and muscle samples, respectively.

Assay of liver triglyceride content in lysate prepared from liver samples from DIO- and lean mice was done using a triglyceride colorimetric assay kit (Cayman Chemical Company, Ann Arbor, MI, USA), according to manufacturer’s instructions.

### Statistical Analysis

Statistical analysis was performed using SAS software version 9.1.3 (SAS Institute Inc., Cary, NC, USA). In all analyses observations were assumed to be independent between animals or cell culture experimental units. Furthermore, data were assumed to follow a normal distribution and to have variance homogeneity. To fulfil these assumptions, data were transformed using the natural logarithm when necessary. Data with a numerical standardized residual value >3.0 were considered as outliers, as suggested previously [41], and the statistical analysis was done with and without these outliers (see further below).

*In vitro* signalling data were analysed using a one-way analysis of variance (ANOVA) followed by pairwise comparisons between each type of treatment and control using multiple t-tests with Dunnetts correction. Furthermore, at each dose level, a direct comparison of treatment with HI and X10 and HI with IGF-1 was done using student t-tests. Data describing IR and IGF-1R expression in various mouse tissues were analysed in a one-way ANOVA followed by pairwise comparisons between tissues using multiple t-test with Bonferroni correction.

The effect of treatment on tumor growth in each of the five animal experiments were analysed in a one-way ANOVA followed by pairwise comparison of each treatment with vehicle using multiple t-tests with Dunnetts correction for each of the two tumor growth endpoints; tumor end volume and area under the tumor growth curves (tumor growth AUC). The results of this analysis for each experiment, i.e., mean values for each treatment, 95% confidence intervals and the fold change of each treatment relative to vehicle, are shown in Table 3.

**Table 3:** Tumor growth *in vivo*.

		Tumor end volume (mm^3^)			AUC tumor growth (day × mm^3^)		
Experiment	Treatment	Mean	95% CI	Fold change†	mean	95% CI	Fold change†
A	Vehicle	499.7	[322.6; 676.7]	1.0	2192.1	[1431.8; 3355.6]	1.0
(n = 6)	HI	439.3	[262.3; 616.4]	0.9	1539.6	[1005.8; 2356.9]	0.7
	X10	500.7	[323.6; 677.7]	1.0	1880.9	[1228.7; 2879.3]	0.9
B	Vehicle	320.5	[122.0; 519.0]	1.0	1181.3	[437.6; 1925.6]	1.0
(n = 6)	HI	434.3	[235.9; 632.8]	1.4	1725.4	[981.7; 2469.1]	1.5
	X10	558.4	[359.9; 756.9]	1.7	2167.5	[1423.8; 2911.2]	1.8
C	Vehicle	326.9	[183.5; 470.4]	1.0	1172.6	[724.7; 1620.5]	1.0
(n = 6)	HI	374.5	[231.0; 517.9]	1.1	1309.5	[861.6; 1757.4]	1.1
	X10	646.8	[503.4; 790.3]	2.0*	2060.9	[1613.0; 2508.8]	1.8*
	IGF-1	724.6	[569.6; 879.5]	2.2*	2596.8	[2113.1; 3080.6]	2.2**
D	Vehicle	178.5	[120.9; 263.6]	1.0	877.8	[402.3; 1353.3]	1.0
(n = 8–10)	HI	397.7	[269.3; 587.2]	2.2*	1483.0	[1007.5; 1958.5]	1.7
	X10	345.8	[234.2; 510.7]	1.9	1394.4	[918.9; 1870.0]	1.6
	IGF-1	616.0	[398.4; 952.5]	3.5**	2207.1	[1675.5; 2738.8]	2.5*
E	Vehicle	331.6	[224.9; 438.3]	1.0	1269.8	[867.0; 1672.7]	1.0
(n = 10)	HI	365.6	[259.0; 472.3]	1.1	1421.9	[1019.1; 1824.7]	1.1
	X10	427.0	[320.3; 533.7]	1.3	1836.1	[1433.2; 2238.9]	1.4
All	Vehicle	335.0	[262.1; 407.8]	1.0	1257.6	[957.5; 1557.8]	1.0
(i.e., A–E)	HI	409.5	[336.7; 482.3]	1.2	1542.1	[1243.8; 1840.3]	1.2
	X10	500.5	[427.7; 573.3]	1.5*	1851.9	[1553.6; 2150.1]	1.5*
	IGF-1	686.3	[574.3; 798.4]	2.0***	2499.8	[2075.5; 2924.1]	2.0***

95% CI = 95% confidence interval.

† fold change, i.e., mean value for a given treatment expressed relative to the mean value of the vehicle-treated group.

* , ** and *** indicates *P*<0.05, <0.001 and <0.0001 respectively.

To examine treatment-related effects on tumor growth across all five animal experiments, we for each endpoint (tumor end volume and tumor growth AUC) pooled all data in one dataset and analysed them in a mixed linear model with treatment as a fixed explanatory variable and experiment as a random effect. No significant interaction between treatment and experiment was found for the any of the two outcomes (tumor end volume and tumor growth AUC). To further explore the differences between treatments, pairwise comparisons of each type of treatment were done using multiple t-test with Bonferonni correction. No outliers were observed among the 131 observations of tumor end volume, whereas one outlier was identified among the 131 observations of tumor growth AUC. Exclusion of this outlier was necessary to fulfil the assumptions behind the statistical models and did not change the overall result of the analysis. The average tumor end volume and tumor growth AUC for each treatment across all five experiments, including 95% confidence intervals and fold change of each treatment relative to the vehicle-treated group are shown in Table 3.

## Results

### MC38 Cells are Responsive to Insulin, X10 and IGF-1

**Figure 1:**
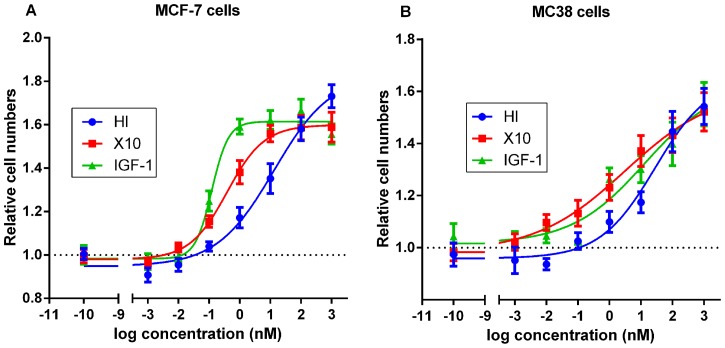
Effect of HI, X10 and IGF-1 on proliferation *in vitro*. MCF-7 cells (A) and MC38 cells (B) were exposed to HI/X10/IGF for 24 h in medium with low serum content (MCF-7; 0.1% (v/v), MC38; 0.25% (v/v)). At the end of the treatment period relative cell numbers were assessed with an MTT assay. Panel A and B shows the mean of three independent experiments, each with four replicates per condition, error bars indicate SEM. HI, X10 and IGF-1 increased proliferation in MCF-7 and MC38 cells. In agreement with previously published data and receptor expression profile, the ranking of the test compounds according to mitogenic potency in MCF-7 cells was IGF-1>X10>HI. In the MC38 cells the ranking was X10≥IGF-1>HI. This suggests IR and IGF-1R are expressed at roughly comparable levels in MC38 cells.

We first examined how treatment with HI, X10 and IGF-1 for 24 h influenced proliferation of MC38 cells and MCF-7 cells (included for comparison), see Figure 1. Treatment with the test compounds increased proliferation in both cell lines. In agreement with previous proliferations studies in MCF-7 cells, and ≈ 9-fold higher expression of IGF-1R than IR in MCF-7 cells [42], the mitogenic effect was largest for IGF-1>X10>HI, whereas in M38 cells the ranking for mitogenic effect was X10≥IGF-1>HI. This suggests MC38 cells express comparable levels of IR and IGF-1R.

**Figure 2:**
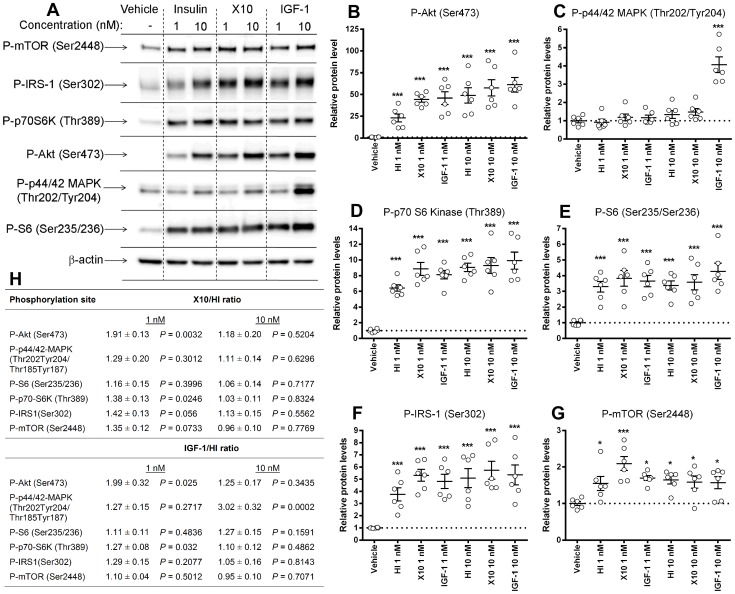
Signalling in MC38 cells after treatment with HI/X10/IGF-1. Representative Western blots are shown on panel A. Cells were harvested after treatment for 30 µg of total protein was loaded on the gels. Each experiment included two replicates per condition. Band intensities were quantified relative to the mean of the two vehicle-treated samples in each experiment. Three independent experiments was performed and results from quantification of band intensities from Western blottting for P-Akt (B), P-p44/42 MAPK (C), P-p70S6K (D), P-S6 (E), P-IRS-1 (F) and P-mTOR (G) and β-actin (load control) were pooled and analyzed in a one-way ANOVA followed by comparison of vehicle-treated samples with each treatment using multiple t-test with Dunetts correction. Finally, a direct comparison of kinase activation by HI and X10 and HI and IGF-1, respectively, was done at each dose level using student t-tests (H). Open circles: one replicate sample, horizontal bars: mean values, error bars: SEM. * and *** indicate *P*<0.05 and *P*<0.0001, respectively.

Furthermore, we examined signalling acutely after treatment with HI, X10 and IGF-1. As shown on Figure 2A–2G, treatment with the chosen doses of HI, X10 and IGF-1 significantly activated IRS-1, Akt, mTOR, p70S6K and S6 in MC38 cells in the phosphoinositide 3-kinase (PI3K) pathway, whereas significant activation of p44/42 mitogen-activated protein kinase (MAPK) was only observed with the high dose of IGF-1 at this time point. A previous study with MCF-7 cells found that P-Akt(Ser473) and P-p70S6K(Ser389) were sensitive endpoints for detection of a signalling difference between HI and X10 [42]. We therefore directly compared equimolar doses of HI, X10 and IGF-1, by calculating the X10/HI and IGF-1/HI ratios for each of the examined kinase phosphorylation sites (Figure 2H). In agreement with the previous study, treatment with X10 significantly increased phosphorylation of Akt(Ser473) and p70S6K(Ser389) at the lowest dose of 1 nM. The signalling data were also in good agreement with the proliferation data, as IGF-1 and X10 appeared equally more potent than HI in stimulating activation of Akt, p70S6K and IRS-1 in MC38 cells.

### Mice with Diet Induced Obesity are Hyperinsulinemic, Glucose-intolerant and have Reduced Insulin Sensitivity

Selected metabolic parameters were measured in DIO-mice and compared to age-matched mice fed a control low fat diet. As shown in Table 2, DIO-mice had approximately 40% increased body weight and were hyperinsulinemic with approximately 4-fold increased insulin levels. However, DIO-mice were only marginally hyperglycemic, as the HbA1C levels were only slightly increased. Chronic exposure to the high fat diet also resulted in hepatic steatosis and DIO-mice had 3- to 4-fold increased levels of triglyceride in the liver. At the functional level, DIO-mice also displayed lower glucose tolerance and had reduced insulin sensitivity, as determined during a glucose tolerance test as described previously [43].

### Treatment with HI, X10 and IGF-1 Results in Short Term but High Systemic Exposure, Decreased Blood Glucose and Suppressed Secretion of Endogenous Insulin

**Figure 3:**
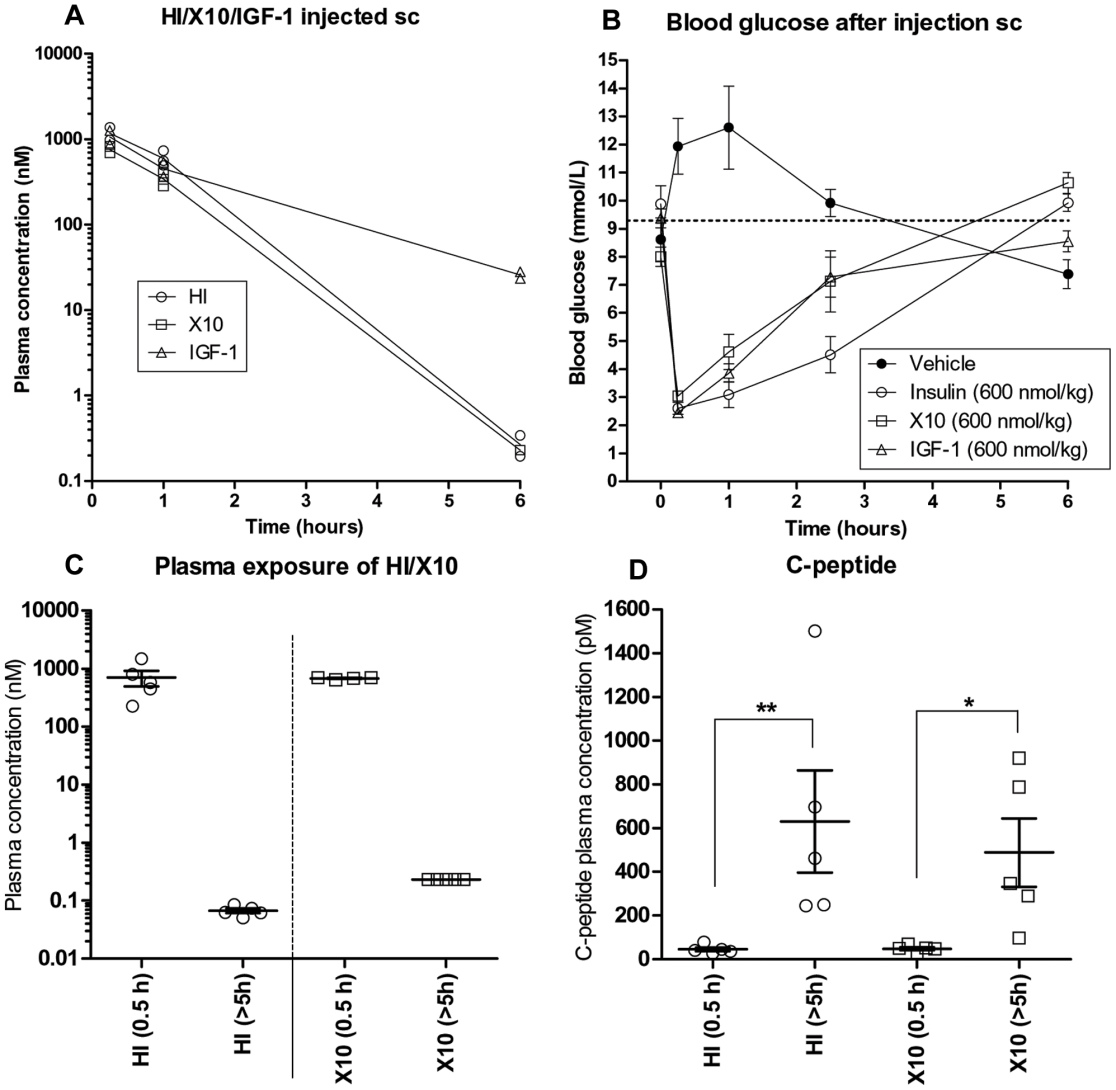
Pharmacokinetics and pharmacodynamics after treatment of DIO-mice with HI, X10 and IGF-1 by sc injection. A: Plasma concentrations measured in mice treated with HI, X10 or IGF-1. The lower detection limits for the different assays were: HI; 2 pM, X10; 233 pM and IGF-1; 1.3 nM. Mice were treated at time 0 and plasma samples were collected after 15 min, 1 h and 6 h. Maximal plasma concentrations were measured 15 min after treatment. 6 h after treatment plasma concentrations of IGF-1 were ≈ 100 fold higher than plasma concentrations of HI and X10. B: Mean blood glucose after treatment with 600 nmol/kg of HI/X10/IGF-1 by injection sc. Blood glucose returned to basal levels after ≈ 3–4 h, n = 8 (HI, X10 and IGF-1) or n = 6 (vehicle). Dotted line indicates mean blood glucose at time 0. Error bars; SEM. C: Plasma concentrations 0.5 h and >5 h after treatment with HI or X10, 600 nmol/kg. Very high plasma concentrations were observed 0.5 h after treatment. Open circles; observations from individual animals, horizontal lines; mean values, error bars; SEM. D: Levels of C-peptide in plasma 0.5 h and >5 h after treatment with HI or X10, 600 nmol/kg, in the same animals as shown on panel C. Levels of C-peptide were significantly decreased 0.5 h after treatment compared to C-peptide levels measured >5 h after sc injection of HI/X10, when blood glucose had returned to basal levels and plasma concentrations of HI and X10 were low. Open circles; observations from individual animals, horizontal lines; mean values, error bars; SEM. * indicate *P*<0.05.

When mice were treated with equimolar supra-pharmacological doses of HI, X10 or IGF-1 by sc injection, very high plasma concentrations were observed shortly after injection (Figure 3A). The C_max_ was approximately 1000 nM for HI, X10 and IGF-1. This is approximately 1000-fold higher than plasma concentrations of endogenous mouse insulin in hyperinsulinemic DIO-mice (Table 2). Based on the plasma concentrations measured 15 min, 1 h and 6 h after sc injection of HI, X10 or IGF-1 (Figure 3A), we could estimate that the plasma elimination half-life (t_½_) for HI and X10 was approximately 30 min, whereas t_½_ for IGF-1 was approximately 70 min, in reasonable agreement with existing literature data and the fact that the majority of IGF-1 in blood plasma is bound to IGF-1 binding proteins [44], [45].

Treatment with the chosen doses of HI, X10 and IGF-1 rapidly lowered blood glucose in the mice in a comparable manner, but approximately 3–4 h after injection blood glucose had returned to basal levels (Figure 3B).

As expected, the levels of C-peptide were low shortly after treatment with HI/X10, where very high plasma exposure was observed (Figure 3C), but as the plasma concentrations of HI/X10 decreased, levels of C-peptide rapidly returned to basal levels >5 h after injection (Figure 3D). This pattern of C-peptide levels also correlated excellently with the changes in blood glucose (Figure 3B).

### Treatment with HI, X10 and IGF-1 Activates Signalling Downstream of IR/IGF-1R in MC38 Cell Allografts *in vivo*

**Figure 4:**
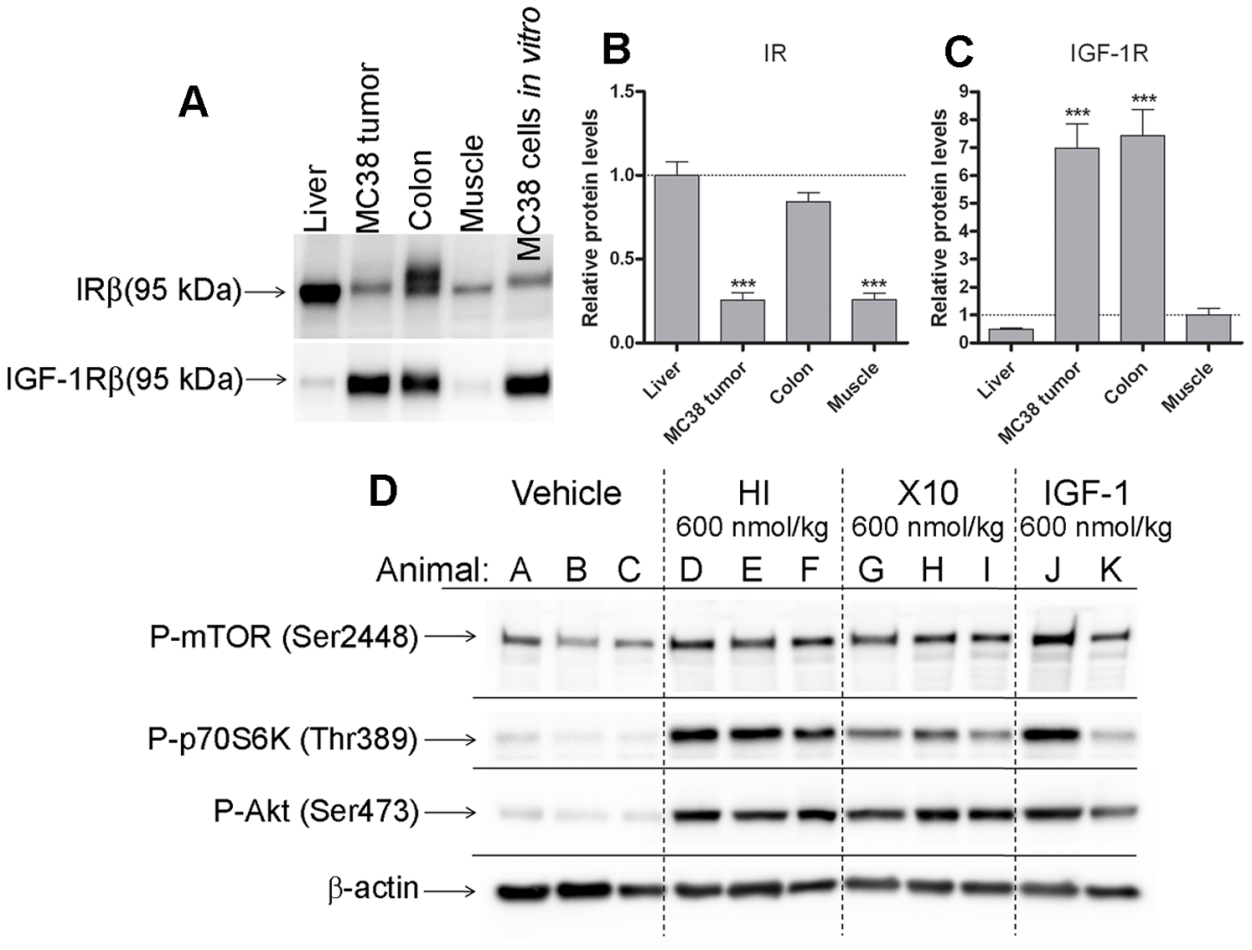
Expression of IR and IGF-1R in MC38 cell allografts and reference tissues. A: Representative Western blots for IRβ and IGF-1Rβ in liver, MC38 tumor, colon, muscle and MC38 cells cultured *in vitro*. For each sample 20 µg of total protein was loaded on the gel. B: Quantitation of Western blots for IRβ was done relative to the average band intensities of liver samples. The relative IR level in MC38 cell allografts was comparable to skeletal muscle and significantly lower than liver and colon. n = 3 or 4 per tissue, bars indicate mean of two experiments, error bars; SEM. *** indicate *P*<0.0001. C: Quantitation of Western blots for IGF-1Rβ was done relative to the average band intensities of muscle samples. The relative IGF-1R level was significantly higher in MC38 cell allografts than muscle and liver and comparable to colon. n = 3 or 4 per tissue, bars indicate mean of two experiments, error bars; SEM. *** indicate *P*<0.0001. D: Representative Western blots for P-mTOR, P-p70S6K, P-Akt and β-actin in samples of tumor allografts collected 1 h after sc injection of HI, X10 or IGF-1. Plasma concentrations of HI, X10 and IGF-1 in these animals at time of euthanasia and collection of tissue samples are shown in Figure 3A. Treatment with HI, X10 or IGF-1 resulted in activation of several kinases in the PI3K signalling pathway.

Expression of IR and IGF-1R in MC38 cell allografts was measured relative to reference tissues liver and skeletal muscle (the gastrocnemius muscle) and normal colon. Both IR and IGF-1R were expressed in MC38 allografts and comparable to MC38 cells cultured *in vitro*, i.e., the phenotype was maintained *in vivo*. In MC38 allografts IR was expressed at lower levels than in liver and colon, but comparable to skeletal muscle. IGF-1R was expressed at levels comparable to colon and at significantly higher levels than liver and skeletal muscle (Figures 4A–4C). With this technique it was no possible to directly compare the levels of IR and IGF-1R between tissues.

We also examined the functionality of the IR and IGF-1Rs in the tumor allografts by Western blotting for kinases in the PI3K signalling pathway in tumor tissue collected 1 h after sc administration of HI, X10 or IGF-1 (Figure 4D). This time point is close to the maximal plasma concentration and the time of maximal effect of the administered compounds on blood glucose. We have previously shown that phosphorylation of Akt in tumor allografts and normal colon after sc injection of a bolus of HI and X10 is strongly time-dependent [36]. In agreement with these data, we observed pronounced activation of Akt, p70S6K and mTOR 1 h after treatment, which demonstrates MC38 tumor allografts are sensitive to acute treatment with HI, X10 and IGF-1.

### Growth of Tumor Allografts is Significantly Increased in Animals Treated with X10 and IGF-1

**Figure 5:**
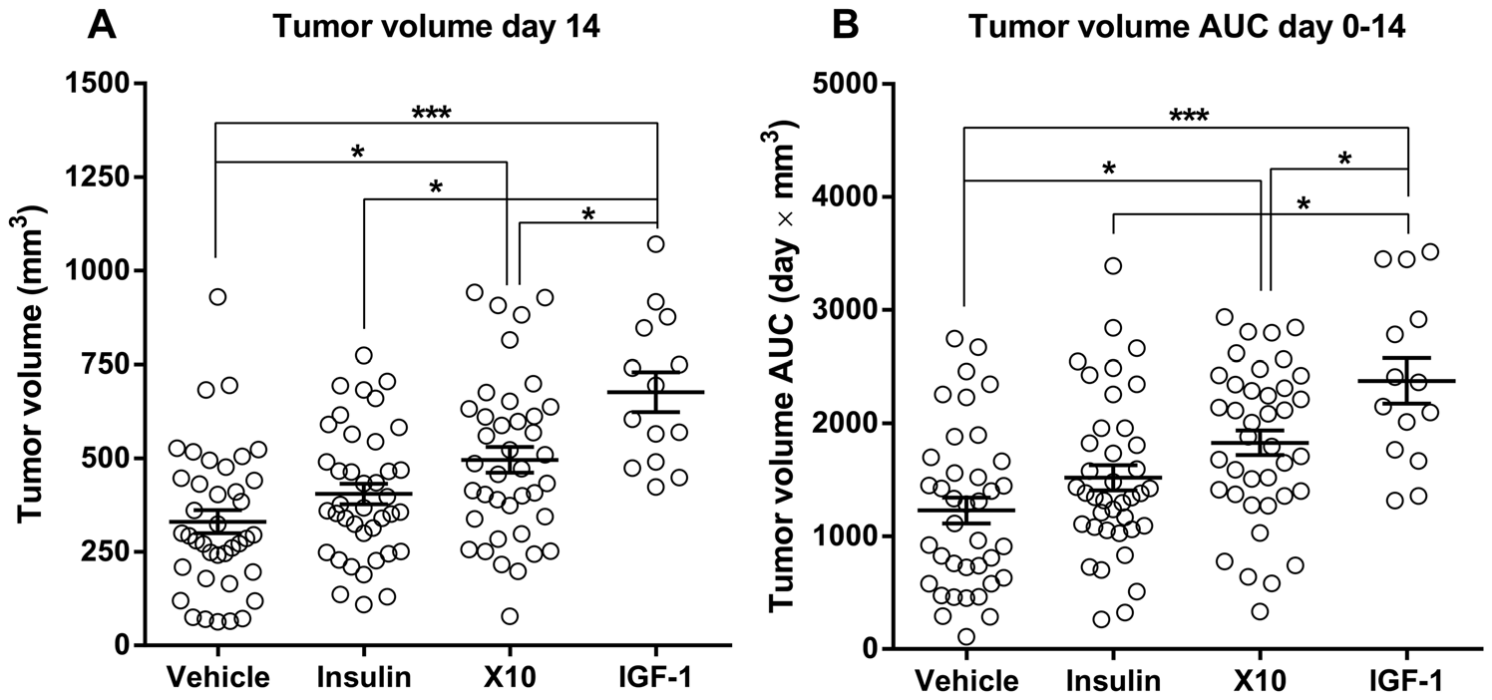
Effect of treatment with HI/X10/IGF-1 on tumor growth. A: Data for tumor volume day 14 in experiment A–E. Treatment with IGF-1 significantly increased tumor volume compared to all other treatments and treatment with X10 increased tumor growth compared to vehicle. B: Area under the tumor growth curves day 0–14 in experiment A–E. These data were in excellent agreement with the data describing tumor volume at day 14; treatment with IGF-1 increased tumor growth compared to all other treatments and treatment with X10 increased tumor growth compared to the vehicle-treated group. Open circles: observations from individual animals, horizontal bars: group mean, error bars: SEM. * and *** indicates *P*<0.05 and 0.0001, respectively.

Five animal experiments were performed. The mean size of tumors at experimental day 14 and tumor growth AUC (experimental day 0 to 14) for each treatment group in each experiment, including 95% confidence intervals for these mean values, are shown in Table 3 and all data are plotted on Figure 5A–B. Except for experiment A, a trend towards increased tumor growth after treatment with HI, X10 and IGF-1 was observed in all experiments, and the fold change in tumor growth were generally of approximately similar magnitude for each type of treatment. However, with the present group sizes and level of variation, these trends were not statistically significant in all experiments. However, when data from all experiments were analyzed in a statistical model where the effect of individual experiments was taken into consideration (see statistics section), treatment with IGF-1 significantly increased tumor volume at day 14 compared to vehicle (≈ 2-fold, *P*<0.0001), HI (*P* = 0.0011) and X10 (*P* = 0.0377). Furthermore, treatment with X10 significantly increased tumor growth compared to vehicle (≈1.5-fold, *P* = 0.0060), but not HI (*P* = 0.2919). No significant difference was observed between vehicle and HI (≈1.2-fold increased, *P* = 0.6092) (Figure 5 and Table 3). The effects of the different treatments are based on five animal experiments and are therefore very robust and reliable estimates of the effect.

In previous studies, tumor volume has been analyzed for each experimental day and/or tumor volume at the end of an experiment has been used as endpoint. We analyzed both tumor volume at day 14 (end of experiments) and tumor growth AUC (day 0 to 14). One could speculate that area under the tumor growth curves would be a more sensitive endpoint than tumor volume at the end of an experiment, since tumor growth AUC comprises information about all measurements of tumor volume during the experiment. However, the two endpoints appeared equally good for distinguishing between treatment-related effects on tumor growth.

## Discussion

Here we show that the MC38 cell line, derived from a murine colon cancer, expresses IRs and IGF-1Rs and is insulin sensitive, as treatment with HI, X10 and IGF-1 results in activation of the PI3K and MAPK signalling pathways and increases proliferation. Treatment of DIO-mice with HI, X10 and IGF-1 also activates the PI3K pathway in MC38 cell allografts and after treatment with X10 and IGF-1 for 14 days, growth of the tumor allografts was significantly increased ≈ 1.5-fold and ≈ 2-fold, respectively, compared to control. This represents an innovative demonstration of growth-promoting effects of X10 and IGF-1 on neoplasms in short-term experiments using obese and hyperinsulinemic animals.

While it might be possible to optimize our experimental design to increase sensitivity, our present results show X10 overall appear to have a weak mitogenic effect on MC38 cell allografts. Despite the animals were treated with very high doses, tumor growth among X10-treated animals was only approximately 1.5-fold increased on average, and due to a considerable variation it was necessary to include a large number of animals to reach statistical significance. Further studies are needed to clarify if this effect is also observed during other experimental conditions. However, our *in vivo* data for tumor growth after treatment with HI and X10 are consistent with the *in vitro* signalling and proliferation data, which show X10 is only a slightly more potent stimulator of proliferation and signalling downstream of IR/IGF-1R than HI in MC38 cells. However, studies with other cell lines have shown a clear difference in mitogenic potency between HI and X10; for some cell lines the mitogenic potency of X10 was 10-fold higher than HI [10], [13], [46]. Our approach to study the stimulatory effect of HI and X10 on neoplastic cells *in vivo* could therefore be extended to include additional cancer cell lines, including those where larger differences between X10 and HI would be expected on the basis of *in vitro* data, to further clarify how mitogenic effects measured *in vitro* correlate with growth stimulatory effects measured *in vivo*.

The dosing regimen in our experiments resulted in transient exposure to high levels of administered insulin together with a simultaneous and temporary suppression of endogenous insulin secretion. We used these supra-pharmacological doses of insulin, X10 and IGF-1 because the increased tumor incidence in previous studies was observed after treatment with supra-pharmacological doses of X10 [14], [15]. Furthermore, supra-pharmacological dose levels are required during preclinical safety assessment of insulin analogs [30]. Our results are important because they show that an increased growth-promoting effect of X10 after short-term treatment with a supra-pharmacological dose is correlated with an increased tumor incidence after long-term treatment with supra-pharmacological doses of X10. In future studies it would be relevant to examine how constant exposure to lower, more clinical relevant, doses of insulin and insulin analogs would influence tumor growth in our short-term model.

In chronic toxicity studies with recombinant human IGF-1, supra-pharmacological doses increased the incidence of mammary carcinoma and pheochromocytoma in female and male rats [47]. In agreement with this, we observed significantly increased growth of MC38 cell allografts in mice treated with IGF-1. In fact, treatment with IGF-1 stimulated tumor growth significantly more than treatment with HI and X10 (Figure 5). This is in contrast to the *in vitro* results, where IGF-1 and X10 appeared equally potent in stimulating proliferation and signalling in MC38 cells (Figure 2). However, the *in vitro* results are based on a single treatment with HI, X10 and IGF-1, whereas the animals were treated with HI, X10 or IGF-1 repeatedly two times daily for 14 days. Furthermore, there are important differences in the pharmacokinetics for HI, X10 and IGF-1; IGF-1 binds to IGF-1 binding proteins and the plasma half-life of IGF-1 is therefore longer than the plasma half-lives for X10 and HI (Figure 3A). Treatment with IGF-1 therefore resulted in exposure for longer time than treatment with HI and X10 (Figure 3A), which could explain the increase in tumor growth following treatment with IGF-1 *in vivo*.

Despite detailed characterization of receptor binding and receptor activation characteristics the mechanism(s) that results in an increased mitogenic effect of X10 are not clear. It has been hypothesized that increased IGF-1R binding affinity will result in an increased mitogenic effect via increased IGF-1R activation. However, a recent study demonstrated that both increased IR binding affinity and increased IGF-1R binding affinity correlate with increased proliferation *in vitro*, in cells with dominant IR or IGF-1R expression, respectively [13]. X10 is characterized by both increased IGF-1R and IR binding affinity and decreased off-rate from the IR [10]–[12], and it is therefore difficult to conclude whether one or all of these characteristics gives X10 an increased growth-stimulatory potential *in vivo*. However, a recent study showed that neither HI or X10 (low to supra-pharmacological dose levels) activates the IGF-1R in different rat tissues [48], which suggests the stronger growth-stimulating effect of an equimolar dose of X10 *in vivo* is mediated via increased IR binding affinity and decreased off-rate from the IR.

Downstream of receptor activation much less is known about growth-promoting effects of X10. We recently demonstrated that treatment of DIO-mice with supra-pharmacological doses of HI and X10 resulted in increased expression of genes in the serine synthesis pathway in MC38 cell allografts, and that treatment of MC38 cells with HI and X10 *in vitro* also resulted in increased synthesis of serine from glucose (X10 more potently than HI) [36]. Although other cellular functions were also affected, stimulation of carbon flux via the serine synthesis pathway is conceptually interesting, as it shows that stimulation of metabolic pathways is linked to stimulation of growth. Another recent study showed that X10 is significantly stronger than HI in phosphorylating several kinases involved in activation of translation, e.g., Akt, p70S6K and S6 [42]. In MC38 cells we observed that X10 significantly increased phosphorylation of Akt (Ser473) and p70S6K (Thr389) with ≈ 35–90% compared to HI, supporting that X10 also in MC38 cells has a stronger activating effect on translation. Stimulation of translation via activation of Akt, mTOR and p70S6K is important for growth of neoplastic cells [49]–[51], and one could speculate that the stronger stimulus by X10 confers a growth advantage to the cells, via increased protein synthesis. It has also been proposed that the stimulatory effect of PI3K and Akt on translation is not simply a general stimulation of protein synthesis, but restricted to translation of specific mRNAs involved in growth control [49].

In conclusion, we have developed a model for evaluating the effect of administered insulins on the growth of pre-existing cancers in insulin resistant animals and demonstrated that supra-pharmacological doses of insulin analog X10 and human IGF-1, hypothesized to be positive control compounds, increase growth of MC38 cell allografts in short-term animal experiments. The clinical relevance of our finding of increased tumor growth after treatment with X10 is unknown. To fully characterise tumor growth-promoting effects of X10 in comparison to HI *in vivo* it is necessary to further explore the effect of doses and pharmacokinetics on growth of allografts, and also to test other cell lines derived from obesity- and diabetes-relevant cancers and/or with a different ratio between IGF-1Rs and IRs in allograft experiments.

## Supporting Information

Figure S1 **Linearity of MTT assays.** By doing an MTT assay (see Materials S1) on newly plated and attached MC38 cells, we confirmed the linear association between number of cells and absorbance measured at 570 nm.
(TIF)

Materials S1 **Supplementary materials and methods.** Detailed description of assays used to measure concentrations of mouse insulin, C-peptide, HI, X10 and IGF-1 in mouse plasma and detailed description of *in vitro* cell proliferation assay.
(DOC)

## References

[1] Adami HO, McLaughlin J, Ekbom A, Berne C, Silverman D, et al (1991) Cancer risk in patients with diabetes mellitus. Cancer Causes Control 2: 307–314. 1932543 10.1007/BF00051670

[2] Calle EE, Kaaks R (2004) Overweight, obesity and cancer: epidemiological evidence and proposed mechanisms. Nat Rev Cancer 4: 579–591 10.1038/nrc1408 [doi];nrc1408 [pii] 15286738

[3] Chen J (2011) Multiple signal pathways in obesity-associated cancer. Obes Rev 12: 1063–1070 10.1111/j.1467-789X.2011.00917.x [doi] 22093240

[4] Coughlin SS, Calle EE, Teras LR, Petrelli J, Thun MJ (2004) Diabetes mellitus as a predictor of cancer mortality in a large cohort of US adults. Am J Epidemiol 159: 1160–1167 10.1093/aje/kwh161 [doi];159/12/1160 [pii] 15191933

[5] Ogunleye AA, Ogston SA, Morris AD, Evans JM (2009) A cohort study of the risk of cancer associated with type 2 diabetes. Br J Cancer 101: 1199–1201 6605240 [pii]; 10.1038/sj.bjc.6605240 [doi] 19690547 PMC2768085

[6] Hemkens LG, Grouven U, Bender R, Gunster C, Gutschmidt S, et al (2009) Risk of malignancies in patients with diabetes treated with human insulin or insulin analogues: a cohort study. Diabetologia 52: 1732–1744 10.1007/s00125-009-1418-4 [doi] 19565214 PMC2723679

[7] Smith U, Gale EA (2009) Does diabetes therapy influence the risk of cancer? Diabetologia 52: 1699–1708 10.1007/s00125-009-1441-5 [doi] 19597799

[8] Gerstein HC, Bosch J, Dagenais GR, Diaz R, Jung H, et al (2012) Basal insulin and cardiovascular and other outcomes in dysglycemia. N Engl J Med 367: 319–328 10.1056/NEJMoa1203858 [doi] 22686416

[9] Brange J, Ribel U, Hansen JF, Dodson G, Hansen MT, et al (1988) Monomeric insulins obtained by protein engineering and their medical implications. Nature 333: 679–682 10.1038/333679a0 [doi] 3287182

[10] Kurtzhals P, Schaffer L, Sorensen A, Kristensen C, Jonassen I, et al (2000) Correlations of receptor binding and metabolic and mitogenic potencies of insulin analogs designed for clinical use. Diabetes 49: 999–1005. 10866053 10.2337/diabetes.49.6.999

[11] Slieker LJ, Brooke GS, DiMarchi RD, Flora DB, Green LK, et al (1997) Modifications in the B10 and B26–30 regions of the B chain of human insulin alter affinity for the human IGF-I receptor more than for the insulin receptor. Diabetologia 40 Suppl 2S54–S61. 9248702 10.1007/s001250051402

[12] Hansen BF, Danielsen GM, Drejer K, Sorensen AR, Wiberg FC, et al (1996) Sustained signalling from the insulin receptor after stimulation with insulin analogues exhibiting increased mitogenic potency. Biochem J 315 (Pt 1): 271–279. 10.1042/bj3150271 PMC1217182 8670118

[13] Hansen BF, Glendorf T, Hegelund AC, Lundby A, Lutzen A, et al (2012) Molecular characterisation of long-acting insulin analogues in comparison with human insulin, IGF-1 and insulin X10. PLoS One 7: e34274 10.1371/journal.pone.0034274 [doi];PONE-D-11-22279 [pii] 22590494 PMC3348127

[14] Gammeltoft S, Hansen BF, Dideriksen L, Lindholm A, Schaffer L, et al (1999) Insulin aspart: a novel rapid-acting human insulin analogue. Expert Opin Investig Drugs 8: 1431–1442 10.1517/13543784.8.9.1431 [doi] 15992160

[15] Dideriksen LH, Jorgensen LN, Drejer K (1992) Carcinogenic effects on female rats after 12 months administration of insulin analog b10 asp. Diabetes 41: 143A.

[16] Hansen BF, Kurtzhals P, Jensen AB, Dejgaard A, Russell-Jones D (2011) Insulin X10 revisited: a super-mitogenic insulin analogue. Diabetologia 54: 2226–2231 10.1007/s00125-011-2203-8 [doi] 21633908

[17] Koohestani N, Chia MC, Pham NA, Tran TT, Minkin S, et al (1998) Aberrant crypt focus promotion and glucose intolerance: correlation in the rat across diets differing in fat, n-3 fatty acids and energy. Carcinogenesis 19: 1679–1684. 9771941 10.1093/carcin/19.9.1679

[18] Tran TT, Gupta N, Goh T, Naigamwalla D, Chia MC, et al (2003) Direct measure of insulin sensitivity with the hyperinsulinemic-euglycemic clamp and surrogate measures of insulin sensitivity with the oral glucose tolerance test: correlations with aberrant crypt foci promotion in rats. Cancer Epidemiol Biomarkers Prev 12: 47–56. 12540503

[19] Lee WM, Lu S, Medline A, Archer MC (2001) Susceptibility of lean and obese Zucker rats to tumorigenesis induced by N-methyl-N-nitrosourea. Cancer Lett 162: 155–160. S0304383500006352 [pii]. 10.1016/s0304-3835(00)00635-2 11146220

[20] Weber RV, Stein DE, Scholes J, Kral JG (2000) Obesity potentiates AOM-induced colon cancer. Dig Dis Sci 45: 890–895. 10795750 10.1023/a:1005560621722

[21] Algire C, Zakikhani M, Blouin MJ, Shuai JH, Pollak M (2008) Metformin attenuates the stimulatory effect of a high-energy diet on in vivo LLC1 carcinoma growth. Endocr Relat Cancer 15: 833–839 ERC-08-0038 [pii]; 10.1677/ERC-08-0038 [doi] 18469156

[22] Algire C, Amrein L, Zakikhani M, Panasci L, Pollak M (2010) Metformin blocks the stimulative effect of a high-energy diet on colon carcinoma growth in vivo and is associated with reduced expression of fatty acid synthase. Endocr Relat Cancer 17: 351–360 ERC-09-0252 [pii]; 10.1677/ERC-09-0252 [doi] 20228137

[23] Ferguson RD, Novosyadlyy R, Fierz Y, Alikhani N, Sun H, et al (2012) Hyperinsulinemia enhances c-Myc-mediated mammary tumor development and advances metastatic progression to the lung in a mouse model of type 2 diabetes. Breast Cancer Res 14: R8 bcr3089 [pii]; 10.1186/bcr3089 [doi] 22226054 PMC3496123

[24] Fierz Y, Novosyadlyy R, Vijayakumar A, Yakar S, LeRoith D (2010) Insulin-sensitizing therapy attenuates type 2 diabetes-mediated mammary tumor progression. Diabetes 59: 686–693 db09–1291 [pii];–10.2337/db091291 [doi] 19959755 PMC2828655

[25] Novosyadlyy R, Lann DE, Vijayakumar A, Rowzee A, Lazzarino DA, et al (2010) Insulin-mediated acceleration of breast cancer development and progression in a nonobese model of type 2 diabetes. Cancer Res 70: 741–751 0008–5472.CAN-09-2141 [pii]; 10.1158/0008-5472.CAN-09-2141 [doi] 20068149 PMC2946167

[26] Tran TT, Medline A, Bruce WR (1996) Insulin promotion of colon tumors in rats. Cancer Epidemiol Biomarkers Prev 5: 1013–1015. 8959325

[27] Heuson JC, Legros N, Heimann R (1972) Influence of insulin administration on growth of the 7,12-dimethylbenz(a)anthracene-induced mammary carcinoma in intact, oophorectomized, and hypophysectomized rats. Cancer Res 32: 233–238. 5058184

[28] Tran TT, Naigamwalla D, Oprescu AI, Lam L, McKeown-Eyssen G, et al (2006) Hyperinsulinemia, but not other factors associated with insulin resistance, acutely enhances colorectal epithelial proliferation in vivo. Endocrinology 147: 1830–1837 en.2005-1012 [pii]; 10.1210/en.2005-1012 [doi] 16410309

[29] Nagel JM, Staffa J, Renner-Muller I, Horst D, Vogeser M, et al (2010) Insulin glargine and NPH insulin increase to a similar degree epithelial cell proliferation and aberrant crypt foci formation in colons of diabetic mice. Horm Cancer 1: 320–330 10.1007/s12672-010-0020-z [doi] 21761363 PMC10358046

[30] EMEA/CPMP (2001) Points To Consider Document on the Non-clinical Assessement of the Carcinogenic Potential of Insulin Analouges. CPMP/SWP/372/01.

[31] Stammberger I, Bube A, Durchfeld-Meyer B, Donaubauer H, Troschau G (2002) Evaluation of the carcinogenic potential of insulin glargine (LANTUS) in rats and mice. Int J Toxicol 21: 171–179 10.1080/10915810290096306 [doi] 12055018

[32] Tennekes H, Gembardt C, Dammann M, van RB (2004) The stability of historical control data for common neoplasms in laboratory rats: adrenal gland (medulla), mammary gland, liver, endocrine pancreas, and pituitary gland. Regul Toxicol Pharmacol 40: 18–27 10.1016/j.yrtph.2004.04.003 [doi];S0273230004000339 [pii] 15265603

[33] Hansen BF (2008) Insulin analogues with increased mitogenic potency–are they safe? Horm Metab Res 40: 431–433 10.1055/s-2008-1062740 [doi] 18418813

[34] Frazier AL, Colditz GA, Fuchs CS, Kuntz KM (2000) Cost-effectiveness of screening for colorectal cancer in the general population. JAMA 284: 1954–1961. joc90997 [pii]. 10.1001/jama.284.15.1954 11035892

[35] Tsoi KK, Ng SS, Leung MC, Sung JJ (2008) Cost-effectiveness analysis on screening for colorectal neoplasm and management of colorectal cancer in Asia. Aliment Pharmacol Ther 28: 353–363 APT3726 [pii];–10.1111/j.13652036.2008.03726.x [doi] 18638075

[36] Hvid H, Fendt SM, Blouin MJ, Birman E, Voisin G, et al (2012) Stimulation of MC38 tumor growth by insulin analog X10 involves the serine synthesis pathway. Endocr Relat Cancer 19: 557–574 ERC-12-0125 [pii]; 10.1530/ERC-12-0125 [doi] 22685267

[37] Poulsen F, Jensen KB (2007) A luminescent oxygen channeling immunoassay for the determination of insulin in human plasma. J Biomol Screen 12: 240–247 1087057106297566 [pii]; 10.1177/1087057106297566 [doi] 17259593

[38] Poulsen F (2012) Wash-LOCI – A Semi-Heterogeneous Version of the LOCI Technology Allowing Removal of Unbound Material After Each Assay Step. In: Abuelzein E, editors. Trends in Immunolabelled and Related Techniques. INTECH. 259–274.

[39] Corbett TH, Griswold DP Jr, Roberts BJ, Peckham JC, Schabel FM Jr (1975) Tumor induction relationships in development of transplantable cancers of the colon in mice for chemotherapy assays, with a note on carcinogen structure. Cancer Res 35: 2434–2439. 1149045

[40] Blouin MJ, Zhao Y, Zakikhani M, Algire C, Piura E, et al (2010) Loss of function of PTEN alters the relationship between glucose concentration and cell proliferation, increases glycolysis, and sensitizes cells to 2-deoxyglucose. Cancer Lett 289: 246–253 S0304-3835(09)00537-0 [pii]; 10.1016/j.canlet.2009.08.021 [doi] 19744772

[41] Bibby BM, Martinussen T, Skovgaard IM (2004) Experimental Design in the Agricultural Sciences. Copenhagen: Samfundslitteratur.

[42] Oleksiewicz MB, Bonnesen C, Hegelund AC, Lundby A, Holm GM, et al (2011) Comparison of intracellular signalling by insulin and the hypermitogenic AspB10 analogue in MCF-7 breast adenocarcinoma cells. J Appl Toxicol 31: 329–341 10.1002/jat.1590 [doi] 20936651

[43] Matsuda M, DeFronzo RA (1999) Insulin sensitivity indices obtained from oral glucose tolerance testing: comparison with the euglycemic insulin clamp. Diabetes Care 22: 1462–1470. 10480510 10.2337/diacare.22.9.1462

[44] Hvid H, Fels JJ, Kirk RK, Thorup I, Jensen HE, et al (2011) In situ phosphorylation of Akt and ERK1/2 in rat mammary gland, colon, and liver following treatment with human insulin and IGF-1. Toxicol Pathol 39: 623–640 0192623311406936 [pii]; 10.1177/0192623311406936 [doi] 21558470

[45] Zapf J, Hauri C, Waldvogel M, Froesch ER (1986) Acute metabolic effects and half-lives of intravenously administered insulinlike growth factors I and II in normal and hypophysectomized rats. J Clin Invest 77: 1768–1775 10.1172/JCI112500 [doi] 3711334 PMC370532

[46] Listov-Saabye N, Jensen MB, Kiehr B, Hansen EW, Svendsen JE, et al (2009) MCF-7 human mammary adenocarcinoma cells exhibit augmented responses to human insulin on a collagen IV surface. J Appl Toxicol 29: 470–477 10.1002/jat.1428 [doi] 19338014

[47] FDA (2005) Pharmacology/Toxicology Review and Evaluation, NDA N. 21839.

[48] Tennagels N, Welte S, Hofmann M, Brenk P, Schmidt R, et al (2013) Differences in metabolic and mitogenic signalling of insulin glargine and insulin aspart B10 in rats. Diabetologia 56: 1826–1834 10.1007/s00125-013-2923-z [doi] 23653049 PMC3699703

[49] Bader AG, Kang S, Zhao L, Vogt PK (2005) Oncogenic PI3K deregulates transcription and translation. Nat Rev Cancer 5: 921–929 nrc1753 [pii]; 10.1038/nrc1753 [doi] 16341083

[50] Petroulakis E, Mamane Y, Le BO, Shahbazian D, Sonenberg N (2006) mTOR signaling: implications for cancer and anticancer therapy. Br J Cancer 94: 195–199 6602902 [pii]; 10.1038/sj.bjc.6602902 [doi] 16404421 PMC2361102

[51] Stephens L, Williams R, Hawkins P (2005) Phosphoinositide 3-kinases as drug targets in cancer. Curr Opin Pharmacol 5: 357–365 S1471-4892(05)00083-4 [pii]; 10.1016/j.coph.2005.03.002 [doi] 15963759

[52] Andrikopoulos S, Blair AR, Deluca N, Fam BC, Proietto J (2008) Evaluating the glucose tolerance test in mice. Am J Physiol Endocrinol Metab 295: E1323–E1332 90617.2008 [pii]; 10.1152/ajpendo.90617.2008 [doi] 18812462

