# Supplementary figures and images for: Treatment with Insulin Analog X10 and IGF-1 Increases Growth of Colon Cancer Allografts
Source: PLoS One. 2013 Nov 18;8(11):e79710. doi: 10.1371/journal.pone.0079710 (PMC3832545; doi:10.1371/journal.pone.0079710)

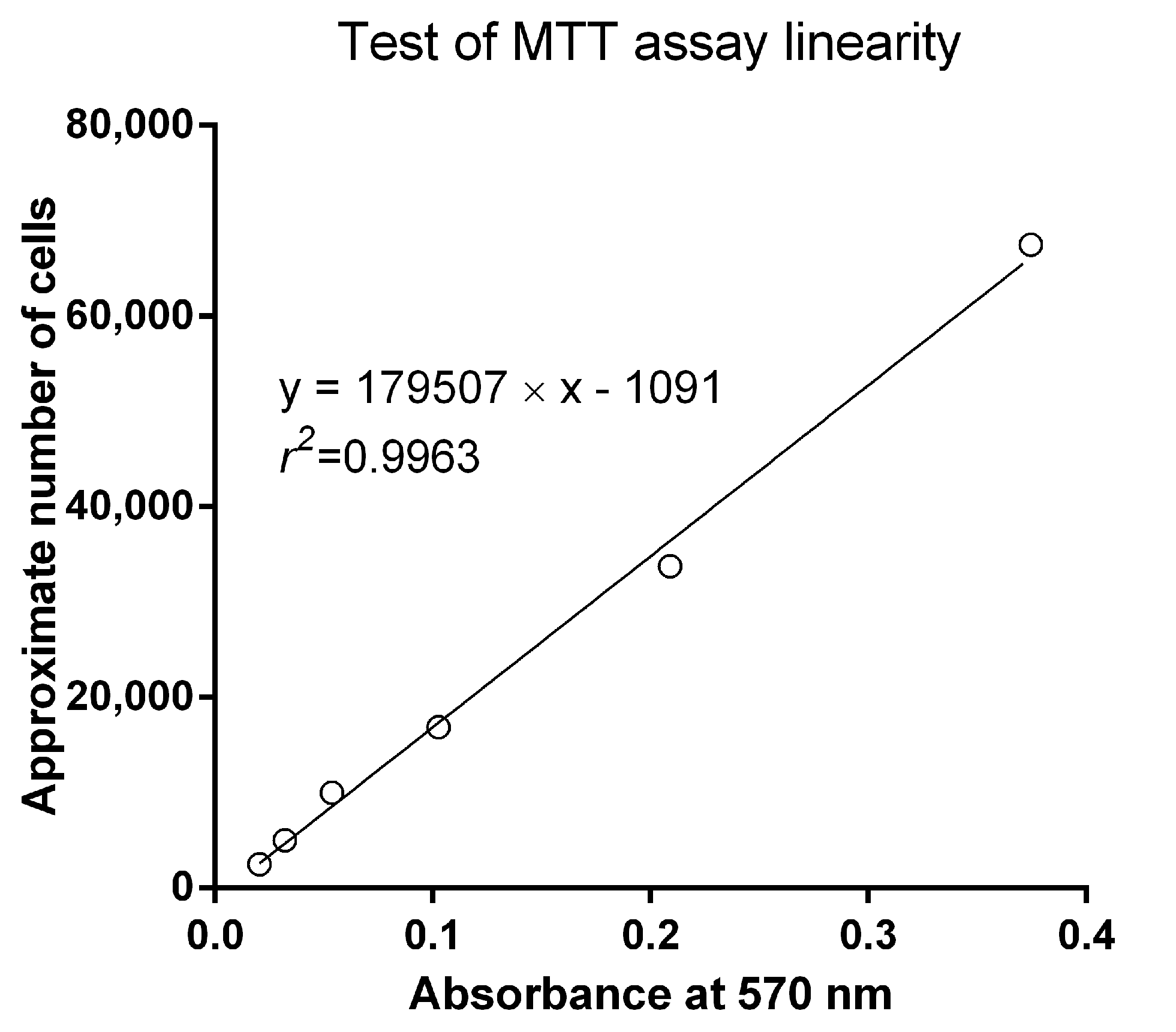

Supplement: Figure S1 — Linearity of MTT assays. By doing an MTT assay (see Materials S1) on newly plated and attached MC38 cells, we confirmed the linear association between number of cells and absorbance measured at 570 nm. (TIF) [file pone.0079710.s001.tif]
