# Supplementary material for: Treatment with Insulin Analog X10 and IGF-1 Increases Growth of Colon Cancer Allografts
Source: PLoS One. 2013 Nov 18;8(11):e79710. doi: 10.1371/journal.pone.0079710 (PMC3832545; doi:10.1371/journal.pone.0079710)
Supplement: Materials S1 — Supplementary materials and methods. Detailed description of assays used to measure concentrations of mouse insulin, C-peptide, HI, X10 and IGF-1 in mouse plasma and detailed description of in vitro cell proliferation assay. (DOC) [file pone.0079710.s002.doc]

**Materials S1**

**Collection of plasma samples and measurements of mouse insulin, C-peptide, human insulin, insulin X10 and human IGF-1**

Before tumor allograft experiments were initiated, blood was collected from all mice to measure the levels of endogenous insulin. Capillary tubes with whole blood were spun at 11700 rpm in a Autocrit Ultra3 centrifuge (BD Scientific, Mississauga, ON, Canada) for 3 min at room temperature and plasma was stored at -80˚C until analysis for mouse insulin using a Rat/Mouse Insulin ELISA kit (Millipore Corp., Bilerica, MA, USA) according to manufacturer’s instructions.

At termination of experiments whole blood was collected in EDTA-coated tubes (BD Biosciences) by cardiac puncture. Blood plasma was isolated by centrifugation for 10 min at 1500 rpm and thereafter stored at -80˚C until analysis. Mouse C-peptide was assayed with Rat/Mouse C-peptide 2 ELISA kit (Millipore) according to manufacturer’s instructions. Plasma concentrations of human IGF-1 were measured with the IDS-iSYS IGF-1 ELISA kit (IDS Immunodiagnostics) according to manufacturer’s instructions. In initial tests the sensitivity and specificity of the assay in mouse plasma was confirmed. Mouse plasma samples were analyzed for native human insulin using a Luminescence Oxygen Channeling Immuno-assay (LOCI-assay) as described previously (Poulsen and Jensen 2007). For the assay, 1 μl sample/standard/control was mixed with beads coated with a mixture of two biotinylated mouse monoclonal anti-insulin antibodies (OXI005 and HUI-018, Novo Nordisk A/S) in 384-well plates at 21-22ºC. After incubation for 1 h at 21–22ºC streptavidin-coated donor beads were added to each well. After incubation for another 30 min at 21-22ºC chemiluminescence was measured in an Envision plate reader (Perkin Elmer). Human insulin diluted in mouse plasma was used as standards. The lower detection limit of the assay was 2 pM. Measurement of X10 in mouse plasma was done in a wash-LOCI assay as described recently (Poulsen 2012). Plate wells were coated with a mouse monoclonal anti-insulin antibody OXI005 (Novo Nordisk A/S) coupled to acceptor beads. Samples and calibrators (mouse plasma) were diluted 1:2 in assay buffer containing 0.015% (w/v) Triton X-100 and 0.05 % (w/v) SDS. Ten µl of this dilution were added to the wells. After incubation for 1 h with gentle shaking at 21-22˚C the plates were washed. A volume of 10 µl biotinylated polyclonal anti-insulin antibody (Fitzgerald) was then added followed by incubation and wash as above. Finally 10 µl of streptavidin coated donor beads were added, incubated as above and immediately after this chemiluminescence was measured using an Envision plate reader. The lower detection limit of the assay was 233 pM.

**Cell proliferation experiments**

In initial tests the association between number of cells and absorbance at 570 nm measured in an MTT assay was examined after incubation of MC38 cells with MTT for 3 h, 3 h after plating different number of cells per well, i.e., the total incubation period was 6 h. As expected there was a highly linear relationship between absolute number of cells and the measured absorbance (Figure S1). To measure *in vitro* mitogenic effects of treatment with HI, X10 and IGF-1 MC38 cells were plated in 96-well plates (5,000 cells per well) in growth medium. After incubation for 1 day cells were rinsed in PBS (room temperature) and then starvation medium was added. After starvation for 3 h cells were treated with HI, X10 or IGF-1 diluted in starvation medium in concentrations ranging from 0.001 to 1000 nM. Control samples were treated with starvation medium only. After treatment for 21 h, 10 µl stock solution of MTT dissolved in PBS (5.0 mg/ml) was added to each well, and incubation was continued for 3 h so treatment with test compounds in total lasted 24 h. At the end of the treatment period medium with test compounds was removed and formazan crystals were suspended by adding 100 µl 0.04 M HCl dissolved in isopropanol to each well and absorbance was thereafter read at 570 nM using a Emax PC plate reader (Molecular Devices LLC, Sunnyvale, CA, USA). Each treatment was done with four replicate samples and three independent experiments were performed. In each experiment the absorbance was normalized to the mean absorbance in the untreated control samples. Data for relative cell numbers from the three repeated experiments were then used to fit dose-response curves using GraphPad Prism version 6.0 (GraphPad Software Inc., La Jolla, CA, USA). Cell proliferation experiments were done with MCF-7 cells as described for MC38 cells, except that 20,000 cells were plated per well. The results from these experiments revealed the same ranking of IGF-1, X10 and HI in respect to mitogenic effect as in previous studies where thymidine incorporation was used to assess mitogenic potency.
